# Comparing the effects of continuous infusion of esmolol and ramosetron alone and in combination on nausea and vomiting after laparoscopic cholecystectomy: A prospective, randomized, double-blind study

**DOI:** 10.1097/MD.0000000000030105

**Published:** 2022-09-02

**Authors:** Jae Young Ji, Nan Seol Kim, Yong Han Seo, Ho Soon Jung, Hea Rim Chun, Jin Soo Park, Jeong Soo Choi, Jae Min Ahn, Woo Jong Kim

**Affiliations:** a Department of Anesthesiology and Pain Medicine, Soonchunhyang University Hospital Cheonan, Chungcheongnam-do, Korea; b Department of Neurosurgery, Soonchunhyang University Hospital Cheonan, Chungcheongnam-do, Korea; c Department of Orthopaedic Surgery, Soonchunhyang University Hospital Cheonan, Chungcheongnam-do, Korea.

**Keywords:** cholecystctomy, esmolol, laparoscopic, ramosetron, postoperative nausea and vomiting

## Abstract

Postoperative nausea and vomiting (PONV) is a common complication of laparoscopic cholecystectomy. Although PONV is usually mild, severe thing can delay recovery and prolong hospitalization. We aimed to investigate the effects of ramosetron and esmolol, alone and in combination, on PONV, and pain.

**Methods:**

We enrolled 165 patients in their 20s to 50s who had an American Society of Anesthesiology physical status score of 1 or 2 and were scheduled to undergo laparoscopic cholecystectomy. They were randomly allocated into 3 groups: groups R, E, and E+R. Patients in group R received 0.3 mg of ramosetron following surgery. Those in group E were intravenously administered a bolus of esmolol (1.0 mg/kg) before endotracheal intubation. They were continuously infused with esmolol during the surgery to maintain their heart rate at 60 to 100 beats per minute and mean blood pressure at 60 to 100 mm Hg, followed by a bolus of esmolol (1.0 mg/kg) following surgery. Patients in group E+R were intravenously administered a bolus of esmolol (1.0 mg/kg) before endotracheal intubation, infused esmolol during surgery, and administered 0.3 mg of ramosetron and a bolus of esmolol (1.0 mg/kg) following surgery. We monitored the PONV stages (none, nausea, retching, and vomiting) and symptom severity in 3 postoperative stages (0–30 minutes, 30 minutes to 6 hours, and 6–24 hours), the latter by using the visual analog scale (VAS). We conducted an analysis of variance to compare VAS scores between groups.

**Results:**

Patients in groups E (mean ± standard deviation VAS score, 3.62 ± 1.00) and E+R (3.66 ± 0.71) exhibited less pain (*P* < .05) until 30 minutes following surgery compared to group R (5.72 ± 1.41). More patients in group E (28/50, 56%) experienced nausea compared to those in groups R (15/50, 30%) and E+R (8/50, 16%) until 30 minutes after surgery (*P* < .05). However, there were no differences in the severity of retching and vomiting between the groups in any of the phases (*P* > .05).

**Conclusion:**

Despite reducing pain after laparoscopic cholecystectomy, esmolol did not prevent PONV, whether used alone or in combination with ramosetron.

## 1. Introduction

Inhalation anesthetics are commonly used to induce general anesthesia. A higher dose of these anesthetics results in a higher risk of side effects, including intraoperative cardiovascular depression, slow recovery, and postoperative respiratory complications. Inhalation anesthetics can also cause complications such as postoperative nausea and vomiting (PONV).^[1,2]^ PONV, a common complication of laparoscopic procedures, reduces patient satisfaction and prolongs hospitalization.^[3,4]^ Several drugs are currently used to prevent PONV; one class of drugs, selective 5-hydroxytryptamine type 3 (5-HT3) receptor antagonists, have seen widespread use, recently.^[5]^

Selective 5-HT3 receptor antagonists are commonly used to prevent vomiting following chemotherapy and are first-line drugs for PONV. This is because they reportedly cause fewer side effects than other antiemetics.^[6]^ Ramosetron, a popular 5-HT3 receptor antagonist, has a stronger receptor affinity and a longer duration of action than other 5-HT3 receptors because of its slow degradation.^[7]^

Esmolol is an ultra-short-acting beta-adrenergic receptor antagonist that selectively affects the cardiovascular system. It reduces sympathetic activity induced by surgical or harmful stimuli, thereby preventing substantial hemodynamic changes. Esmolol-induced hemodynamic stabilization reportedly contributes to the reduction of PONV and postoperative pain.^[8,9]^

Generally, ramosetron is used for the prevention of PONV, while esmolol can help in preventing PONV by providing patient hemodynamic stability during surgeries.^[7,8,10,11]^ In previous studies, various antiemetics have been used in combination to reduce PONV.^[12]^ However, there have been no reports on the effect of the combined use of ramosetron and esmolol on PONV. Therefore, we conducted the study with 3 groups, ramosetron only, esmolol only, and esmolol and ramosetron combination, to assess whether the combination of ramosetron and esmolol has better preventive effects for PONV than ramosetron monotherapy.

## 2. Materials and Methods

**Figure 1.**
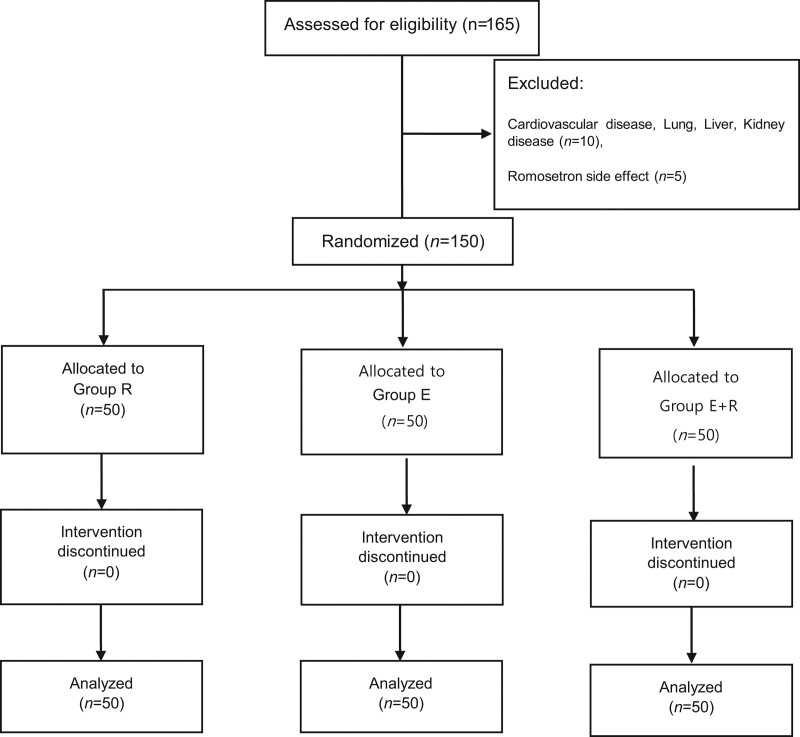
Consort flow diagram of this study.

This prospective, randomized, double-blind study was approved by the Soon Chun Hyang Institutional Review Board (no. 2017-12-018-007). This study was registered in the Clinical Research Information Service (No. KCT0006178), and written informed consent was obtained from each patients. We enrolled 165 patients in their 20s to 50s who had an American Society of Anesthesiology physical status score of 1 or 2 and were scheduled to undergo laparoscopic cholecystectomy. They were allocated into 3 groups using a computerized random sequence generator program (www.random.org): group R, group E, and group E+R. A consort flow diagram is shown in Figure 1. Female sex is considered a risk factor for PONV.^[13]^ Therefore, the patients were divided into the groups with similar sex ratios. We excluded patients who had experienced side effects of esmolol or ramosetron; demonstrated hypersensitivity to esmolol or ramosetron; cardiovascular, lung, or kidney conditions; or neurologic disorders, as well as those who were pregnant (Fig. 1).

Before inducing anesthesia, the patients were monitored by using electrocardiography, a noninvasive blood pressure monitor, a pulse oximeter, and the bispectral index (BIS). The doses of anesthetic agents were controlled such that the BIS value was 40 to 60.^[14]^ Propofol (2 mg/kg) and rocuronium (0.8 mg/kg) were infused to induce anesthesia. Patients in group R were not administered intraoperative esmolol if their heart rate (HR) was <100 beats per minute (bpm) before endotracheal intubation. All patients in group R were administered 0.3 mg of ramosetron postoperatively.^[15]^ Those in group E were intravenously administered a bolus of esmolol (1.0 mg/kg) before endotracheal intubation. In those patients, esmolol was continuously infused to maintain their HR and mean blood pressure (MBP) at 60 to 100 bpm and 60 to 100 mm Hg,^[16]^ respectively, during the surgery. Moreover, they were infused with a bolus of esmolol (1.0 mg/kg) during extubation following the surgery. Patients in group E+R were administered a bolus of esmolol (1.0 mg/kg) before endotracheal intubation. We continuously infused esmolol during the surgery, followed by 0.3 mg of ramosetron postoperatively and a bolus of esmolol (1.0 mg/kg) immediately after extubation. In both groups E and E+R, we stopped the continuous infusion of esmolol as soon as the wound was closed. All 3 groups were administered fentanyl for blood pressure control and postoperative pain management. We administered 1 µg/kg fentanyl, as the risk of PONV increases with opioid use. For patients in all 3 groups in which the blood pressure remained high, nicardipine was administered. Moreover, we administered ephedrine or atropine to control the blood pressure of patients in whom the MBP dropped below 60 mm Hg, and in patients who experienced bradycardia.

Intraperitoneal CO_2_ insufflation was maintained at 12 mm Hg.^[17]^ We intravenously administered a mixture of glycopyrrolate (0.008 mg/kg) and pyridostigmine (0.03 mg/kg) as a reversal agent for all patients. We monitored the PONV stages (none, nausea, retching, and vomiting) and symptom severity in 3 postoperative time stages (0–30 minutes, 30 minutes to 6 hours, and 6–24 hours), the latter by using the visual analog scale (VAS). Nausea was defined as the urge to vomit. Retching was defined as contraction of the abdominal muscles without discharge of the stomach contents. Vomiting was defined as the forceful expulsion of the stomach contents.^[18]^ Ramosetron was administered if the patient had a nausea score of ≥3 following surgery. Ketorolac was administered if the patient had a VAS score ≥3. The dosages of the drugs administered were recorded. Another researcher who did not participate in the study recorded PONV severity and pain in the recovery room.

### 2.1. Statistical analysis

Continuous data are presented as mean ± standard deviation. We conducted an analysis of variance to compare the continuous variables, namely VAS scores, MBP, and HR between the groups. Categorical data are presented as frequencies or percentages (%). A chi-square test or Fisher exact test was used to compare nausea, retching, vomiting, the use of antiemetics, and the use of ketorolac between the groups. The level of statistical significance was set at *P* < .05.

## 3. Results

From May 2018 to 2020 December, the study was conducted on 150 patients. Patients with cardiovascular disease, lung, liver, kidney disease (n = 10) and patients with side effects in ramosetron (n = 5) were excluded from the study (Fig. 1). We observed no differences in age, sex, weight, height, body mass index, duration of surgery or anesthesia, smoking status, history of motion sickness or previous PONV, discharge time, or fentanyl usage between the groups (Table 1). Group R (40.20 ± 13.89 mg) received less esmolol than group E (173.64 ± 30.25 mg) and E+R (168.01 ± 30.06) (*P* < .001). More patients in group E (56%) experienced nausea compared to those in groups R (30%) and E+R (16%) until 30 minutes after surgery (*P* < .001) (Table 2). In addition, group E (56%) demonstrated greater additional ramosetron use than groups R (24%) and E+R (12%) (*P* < .001). However, there were no differences in the severity of retching and vomiting between the groups in any of the phases. Patients in group R (5.72 ± 1.41) reported more severe pain than those in groups E (3.62 ± 1.00) and E+R (3.66 ± 0.71) until 30 minutes after surgery (Table 3). Moreover, patients in group R (54.6 mg) received larger doses of ketorolac than those in groups E (39.0 mg) and E+R (38.4 mg) (*P* < .001). However, we observed no significant differences in pain severity between the groups after patients left the recovery room.

**Table 1:** Patient characteristics.

	Total	Group R	Group E	Group E+R	*P* value
Number of participants	150	50	50	50	
Age (yr)	44.02 ± 9.11	43.76 ± 9.46	43.92 ± 9.51	44.38 ± 8.52	.94
Sex					
Male	47 (31.33%)	15 (30%)	17 (34%)	15 (30%)	.88
Female	103 (68.67%)	35 (70%)	33 (66%)	35 (70%)	
Weight (kg)	67.63 ± 12.74	68.45 ± 13.88	66.81 ± 11.68	67.63 ± 12.78	.81
Height (cm)	163.57 ± 8.42	164.17 ± 8.94	162.69 ± 8.18	163.85 ± 8.22	.66
BMI (kg/m^2^)	25.19 ± 3.48	25.34 ± 3.72	25.09 ± 3.04	25.13 ± 3.71	.93
Duration of surgery (min)	39.13 ± 12.49	41.40 ± 15.81	36.50 ± 12.72	39.50 ± 7.09	.14
Duration of anesthesia (min)	60.95 ± 12.84	62.42 ± 16.31	58.72 ± 13.56	61.70 ± 6.59	.31
Smoking status (n)					
Smoker	38 (25.33%)	12 (24%)	12 (24%)	14 (28%)	.87
Nonsmoker	112 (74.67%)	38 (76%)	38 (76%)	36 (72%)	
History of motion sickness (n)					
Yes	32 (21.33%)	9 (18%)	12 (24%)	11 (22%)	.76
No	118 (78.67%)	41 (82%)	38 (76%)	39 (78%)	
History of previous PONV (n)					
Yes	8 (5.33%)	3 (6%)	1 (2%)	4 (8%)	.53
No	142 (94.67%)	47 (94%)	49 (98%)	46 (92%)	
Perioperative esmolol usage (mg)	127.28 ± 66.96	40.20 ± 13.89	173.64 ± 30.25 *	168.01 ± 30.06 *	**<.001**
Discharge time (hr)	30.87 ± 2.82	31.00 ± 3.03	31.20 ± 3.28	30.40 ± 1.98	.34
Fentanyl usage (µg)	66.59 ± 13.41	65.46 ± 12.70	66.50 ± 13.60	67.80 ± 14.08	.69

Data are presented as mean ± standard deviation or the number of patients.

BMI = body mass index, E = esmolol, PONV = postoperative nausea and vomiting, R = ramosetron.

* Statistically significant difference with group R (*P* < .05).

**Table 2:** The incidence and severity of postoperative nausea and vomiting and ramosetron usage.

	Total	Group R	Group E	Group E+R	*P* value
Number of patients	150	50	50	50	
Nausea					
30 min	51 (34%)	15 (30%)	28 (56%)*	8 (16%)	**<.001**
30 min to 6 hr	20 (13%)	6 (12%)	11 (22%)	3 (6%)	.06
6–24 hr	11 (7%)	3 (6%)	4 (8%)	4 (8%)	>.99
Retching					
30 min	10 (7%)	3 (6%)	5 (10%)	2 (4%)	.61
30 min to 6 hr	2 (1%)	0 (0%)	2 (4%)	0 (0%)	.33
6–24 hr	0 (0%)	0 (0%)	0 (0%)	0 (0%)	>.99
Vomiting					
30 min	4 (3%)	2 (4%)	1 (2%)	1 (2%)	>.99
30 min to 6 hr	0 (0%)	0 (0%)	0 (0%)	0 (0%)	>.99
6–24 h	0 (0%)	0 (0%)	0 (0%)	0 (0%)	>.99
Additional ramosetron use	46 (31%)	12 (24%)	28 (56%)*	6 (12%)	**<.001**

E = esmolol, PONV = postoperative nausea and vomiting, R = ramosetron.

* Statistically significant difference (*P* < .05) with group R.

**Table 3:** Visual analog scale scores and ketorolac use.

	Total	Group R	Group E	Group E+R	*P* value
Number of patients	150	50	50	50	
VAS score					
30 min	4.33 ± 1.45	5.72 ± 1.41	3.62 ± 1.00*	3.66 ± 0.71*	**<.001**
30 min to 6 hr	3.81 ± 1.32	3.42 ± 1.07	3.40 ± 1.34	3.04 ± 0.78	.15
6–24 hr	1.82 ± 0.82	1.90 ± 0.81	1.76 ± 0.98	1.80 ± 0.57	.67
Ketorolac (mg)	44.0	54.6	39.0*	38.4*	**<.001**

Data are presented as mean ± standard deviation.

E = esmolol, R = ramosetron, VAS = visual analog scale.

* Statistically significant difference (*P* < .05) with group R.

Patients in groups E and E+R exhibited a lower MBP compared to those in group R, immediately following intubation (group R: 102.92 ± 9.12 mm Hg, group E: 91.72 ± 9.27 mm Hg, and group E+R: 92.36 ± 6.39 mm Hg) and after extubation (group R: 99.80 ± 8.13 mm Hg, group E: 91.86 ± 7.35 mm Hg, and group E+R: 89.24 ± 5.26 mm Hg) (*P* < .001) (Table 4). Patients in group R exhibited higher HRs than groups E or E+R immediately following intubation (group R: 92.82 ± 11.43 bpm, group E: 83.26 ± 12.70 bpm, and group E+R: 84.16 ± 7.19 bpm; *P* < .001), following incision (group R: 89.00 ± 11.97 bpm, group E: 84.26 ± 12.73 bpm, and group E+R: 83.88 ± 7.53 bpm; *P* = .04), and following extubation (group R: 101.02 ± 8.77 bpm, group E: 91.06 ± 10.25 bpm, and group E+R: 87.24 ± 7.00 bpm; *P* < .001).

**Table 4:** Differences in the mean arterial pressure and heart rate.

	Total	Group R	Group E	Group E+R	*P* value
Number of patients	150	50	50	50	
MBP1† (mm Hg)	89.44 ± 8.28	89.70 ± 10.18	88.60 ± 7.89	90.02 ± 6.46	.67
MBP2‡ (mm Hg)	95.67 ± 9.78	102.9 ± 9.12	91.72 ± 9.27*	92.36 ± 6.39*	**<.001**
MBP3§ (mm Hg)	90.23 ± 15.43	92.70 ± 20.02	88.74 ± 17.16	89.26 ± 4.39	.38
MBP4∥ (mm Hg)	93.63 ± 8.30	99.80 ± 8.13	91.86 ± 7.35*	89.24 ± 5.26*	**<.001**
HR1† (bpm)	81.87 ± 12.64	83.20 ± 14.74	81.90 ± 14.12	80.52 ± 8.08	.57
HR2‡ (bpm)	86.75 ± 11.48	92.82 ± 11.43	83.26 ± 12.70*	84.16 ± 7.19*	**<.001**
HR3§ (bpm)	85.71 ± 11.16	89.00 ± 11.97	84.26 ± 12.73*	83.88 ± 7.53*	**.04**
HR4∥ (bpm)	93.11 ± 10.48	101.02 ± 8.77	91.06 ± 10.25*	87.24 ± 7.00*	**<.001**

Data are presented as mean ± standard deviation.

bpm = beats per minute, E = esmolol, HR = heart rate, MBP = mean blood pressure, R = ramosetron.

* Statistically significant difference (*P* < .05) with group R.

† Measured preinduction.

‡ Measured immediately following intubation.

§ Measured following incision.

∥ Measured following extubation.

## 4. Discussion

PONV is a common side effect reported at a rate of 30% to 80% after surgeries, but it should not be overlooked, as patients may be at discomfort or this could lead to longer hospital stay or readmission.^[19]^

Various drugs, including antihistamines, butyrophenones, and dopamine receptor antagonists, are used to prevent PONV. However, those drugs have side effects such as excessive relaxation, hypotension, dry mouth, unpleasant feelings, and hallucinations in the case of droperidol and metoclopramide.^[20]^

In this study, ramosetron, an antiemetic used during surgeries, and esmolol, for the safety of hemodynamic stability during anesthesia and surgery, were used. The effect of these agents for the prevention of PONV was investigated. In order to best understand the effects of esmolol, its use in Group R was minimized, except for tachycardia during anesthesia or surgery. Patients that were administered esmolol exhibited less pain for up to 30 minutes following surgery than those who were not. However, patients who were administered esmolol only and exhibited no reduction in PONV. Although the number of patients who experienced nausea was lower in group E+R than in group R up to 6 h after surgery, the difference was not statistically significant.

The effect of esmolol is known to increase with the dose; however, administration of a bolus (0.5–4 mg/kg) or infusion (5–500 µg/kg/min) of esmolol reportedly causes unexpected hypotension.^[16]^ Elsewhere, it was reported that continuous administration of esmolol (30 µg/kg) did not cause hypotension or bradycardia.^[21]^ Thus, it is difficult to determine the dose at which side effects are minimized. We achieved a stable HR and blood pressure at a dosage of 10 ± 7 µg/kg/min. Although the hemodynamics against stimulations, such as the intubation and incision, was more stabilized in Group E that received esmolol infusion, Group E did not demonstrate efficacy against PONV comparable to the effects demonstrated in Group R. Increased esmolol dose may demonstrate better prevention against PONV, and therefore future studies with revised esmolol dose may be needed.

Despite limited evidence for the risk factors of PONV, multiple factors have been identified, including age, sex, obesity, a history of motion sickness, previous PONV, operative procedures, anesthetic technique used, and postoperative pain.^[22]^ Laparoscopic cholecystectomy induces PONV.^[13]^ The incidence of PONV following laparoscopic cholecystectomy is 40% to 75% in the absence of prophylactic antiemetics.^[23–25]^ The surgery itself, an increase in abdominal pressure during laparoscopy, and an increase in intracranial pressure due to carbon dioxide absorption, which causes the dilation of cerebral blood vessels, are other possible causes of PONV following laparoscopic cholecystectomy.^[26]^

Because there could be a large hemodynamic fluctuation due to the stimulation from surgery or anesthesia, remifentanil or fentanyl is generally used for safety reasons.^[27]^ However, nausea and vomiting are side effects of opioids, which could impact the results of this study.^[28]^ Therefore, we maintained the dose of fentanyl below 1 µg/kg during surgery and managed patients’ postoperative pain by using ketorolac.

5-HT3 receptor antagonists, including ondansetron, tropisetron, and ramosetron, are also used to prevent PONV. Tropisetron is less effective than ramosetron in preventing PONV. The US Food and Drug Administration recommends administering ondansetron with caution because it can induce QT prolongation. Thus, ramosetron, which is commonly used, was selected for this study.^[13]^

Ramosetron may be administered several times if the initial administration fails to effectively prevent PONV. In several studies, no difference in effectiveness between a 0.3 and a 0.6-mg dose was reported. Hence, additional ramosetron administration would likely be ineffective. However, ramosetron appeared effective since 0.6 mg was administered at a certain time interval.^[29,30]^

There is ample evidence that antiemetics with different mechanisms of action exert a synergistic effect when used in combination to prevent PONV. For example, 5-HT3 receptor antagonists (granisetron, ondansetron, and tropisetron) prevent PONV more effectively when used in conjunction with dexamethasone, metoclopramide, or droperidol.^[31]^ To our knowledge, there are no reports of the use of a single antiemetic drug that can completely eliminate PONV, and that the risk of side effects must be considered when administering a single drug at a high dose. For instance, the frequency of side effects increases with the dose of metoclopramide.^[32]^ Furthermore, administration of metoclopramide in conjunction with dexamethasone at appropriate doses is more effective, safer, and less costly than the use of metoclopramide alone. This necessitates the investigation of the effect of combining those 2 drugs for PONV prevention.^[33]^

While there is sufficient evidence for a sparing effect of esmolol on opioids or anesthetic agents,^[34–36]^ there have been few reported investigations of whether esmolol alone reduces PONV or pain. However, one of the risk factors of PONV is Excessive intraoperative cardiovascular change,^[37]^ and we hypothesized that minimizing the hemodynamic fluctuations provoked by surgeries via the administration of esmolol could prevent PONV. Also, gastric motility is associated with stress, which, in turn, is reduced by beta-blockers. Thus, esmolol may prevent nausea or vomiting.^[38]^ In addition, beta-blockers reportedly exert antinociceptive effects by regulating Ca^2+^ channels. Hence, it was predicted that esmolol would effectively reduce pain.^[32]^ Our findings suggest that the administration of esmolol reduces pain until 30 minutes after surgery, but not PONV.

## 5. Limitations

Our study was subject to several limitations. As there is no clear evidence that esmolol reduces PONV, an assessment of the effects of esmolol on PONV prevention may be unethical when its preventive effect is unclear.

However, since esmolol administration can, mechanistically, reduce blood pressure fluctuations provoked by surgeries, we can anticipate prevention of PONV from this, and patients in group E were immediately administered antiemetics upon experiencing symptoms of PONV in the recovery room. Therefore, the administration of esmolol was deemed unproblematic.

We did not record the dose of ramosetron or ketorolac administered on an hourly basis. However, the 3 groups exhibited differences in nausea and pain only until 30 minutes after surgery; accordingly, the ramosetron or ketorolac doses also differed only until 30 minutes after surgery. Thus, we recorded the doses administered only up to that time point.

The dose of esmolol administered may appear arbitrary. However, there is no definitive standard for esmolol dose.^[39]^ Moreover, we set the dose according to that necessary to maintain a normal blood pressure and HR. Thus, we do not consider the esmolol dose to have affected the results. Further research on the dose of esmolol required to prevent PONV is necessary.

The administration of fentanyl, which can affect postoperative PONV, may be questionable. However, fentanyl is commonly used to manage postoperative pain. Furthermore, it was administered at a dose of 1 µg/kg to control blood pressure in this study, based on a report that a 2-µg/kg bolus of fentanyl can increase the risk of PONV.^[40]^ Thus, we presumed that half that dose of fentanyl would not substantially affect PONV.

## 6. Conclusion

Esmolol reduces pain for up to 30 minutes following laparoscopic cholecystectomy. However, esmolol alone or esmolol, ramosetron combined administration does not prevent PONV. Thus, further research is warranted to determine whether a higher dose of esmolol is able to reduce PONV.

## Author contributions

**Conceptualization:** Jae Young Ji, Nan Seol Kim, Woo Jong Kim.

**Data curation:** Nan Seol Kim, Yong Han Seo, Ho Soon Jung, Hea Rim Chun, Jin Soo Park, Jeong Soo Choi, Jae Min Ahn.

**Investigation:** Jae Young Ji.

**Methodology:** Jae Young Ji.

**Supervision:** Jae Min Ahn.

**Writing – review & editing:** Jae Young Ji, Nan Seol Kim.
